# The Investigation of Volatile Organic Compounds (VOCs) Emissions in Environmentally Friendly Modified Asphalt

**DOI:** 10.3390/polym14173459

**Published:** 2022-08-24

**Authors:** Shuoqiu Chen, Jiaqing Wang, Qiang Li, Wenxuan Zhang, Chaojie Yan

**Affiliations:** College of Civil Engineering, Nanjing Forestry University, Nanjing 210037, China

**Keywords:** environmentally friendly modified asphalt, VOCs suppression effect, optimal modifier combination, asphalt binder

## Abstract

Asphalt pavements are increasingly used in road engineering; however, the release of volatile organic compounds (VOCs) from asphalt can harm the environment and humans. In this study, different types of modifiers are added to 70# virgin asphalt to prepare environmentally friendly modified asphalt, and its performance is analyzed. Through the self-designed simple asphalt heating-emission collection and detection device, the inhibition effect of different types of modifier combinations on VOCs in the asphalt emmissions was explored. Then, VOCs emission curves of modified asphalt at different temperatures were studied, and finally the basic physical properties of the environmentally friendly modified asphalt were tested. The test results showed that the optimal modifier combination was 5% activated carbon and 3% surfactant, in which the VOCs and the peak value of asphalt heating emissions were only 1385 min·ppm and 86 ppm, respectively, which represented the best VOCs suppression effect of other groups. At the same time, the modified asphalt with optimal additives improved the high-temperature performance of 70# base asphalt and did not affect the storage stability.

## 1. Introduction

With the progress of society and the development of science and technology, the living standard of human beings has been gradually improved, and the awareness of environmental protection has also been improved. It has become the consensus of the world to reduce and manage carbon emissions [1]. As of April 2021, the total length of expressways open to traffic in China is 160,000 km, ranking among the top in the world. Asphalt pavement accounts for 90% in China. 70# base asphalt is often used in North, Central and South areas where the summer temperature is high in China. It belongs to heavy traffic petroleum asphalt and can be used for any grade of asphalt pavement, so it is widely used. To reduce the viscosity of the asphalt material and meet the fluidity requirements of the pavement, most asphalt pavement constructions use hot mix asphalt (HMA). However, HMA is continuously heated at high temperature and generates a large number of fumes during manufacturing, transportation, and construction. Typical components of asphalt flue gas include volatile organic compounds (VOCs), polycyclic aromatic hydrocarbons (PAHs), particulate matter (PM), sulfur oxides, nitrogen oxides, and carbon monoxide. Existing literature mainly focuses on volatile organic compounds, PAHs, and particulate matter in bitumen flue gas [2,3]. Especially in asphalt flue gas, the content of volatile organic compounds is the highest and the variety is the most [4,5]. It is widely recognized as a precursor of PM 2.5 and ozone pollution toxicity, with adverse effects on both human health and environmental safety [6,7,8]. As early as 1987, the International Agency for Research on Cancer (IARC) listed bitumen fumes as a suspected carcinogen. In China, in order to protect the health of employees in a polluted environment, the new regulation (GB31570-2015) sets the emission standard for asphalt fume concentration at 20 mg/m^3^ [9,10]. It is worth mentioning that the global demand for bitumen is estimated to be around 143 million metric tons per year (MMTPA) in 2020 and is expected to reach 174 MMTPA by 2025, with bitumen used for road paving accounting for around 85% of its total consumption [11]. Therefore, reducing the emission of asphalt, improving the construction environment of asphalt roads, and reducing the harm to the surrounding environment are important goals to achieve green and sustainable road infrastructure.

Relevant domestic and foreign researchers have added modifiers to suppress the emission of VOCs in heating emissions. According to their mechanism of action, modifiers can be divided into three categories [12]. The first is a polymer modifier, by which the internal structure of the asphalt binder becomes tighter. Asphalt molecules, especially light components, are fixed in the network structure by forming a network structure, thereby reducing the emission of asphalt VOCs [13,14]. Cui et al. [15] studied the inhibition of VOCs by styrene-butadiene-styrene block copolymer (SBS), activated carbon, and layered double hydroxide. The results show that the emission reduction effects are activated carbon, layered double hydroxide, and SBS in order. The addition of SBS can improve the high and low-temperature performance of asphalt materials, but the smoke suppression effect is poor. Sukhija [16] used polymers (e.g., coumarone resin, polyethylene glycol, unsaturated polyester resin, p-benzyl alcohol, and epoxy resin) as cross-linking agents to study the effect of different cross-linking agents and reaction conditions on asphalt VOCs emission reduction effect. The second is the adsorbent, which is divided into physical adsorbents and chemical adsorbents [17,18,19]. The general process of physical adsorption is that the porous structure of the adsorbent immobilizes the macromolecular components. Chemisorption is formed by the shared electron pairs formed by bitumen VOCs and abatement agents. Fine-grained activated carbon is an adsorbent, and the carbonization and activation process at high temperatures contributes to the formation of activated carbon pore structure. Due to its porous structure and huge specific surface area, activated carbon has a high adsorption capacity [20,21], showing a good adsorption effect on asphalt VOCs. Zhou et al. [22] used biochar to adsorb pitch VOCs and investigated the adsorption mechanism by pyrolysis gas chromatography-mass spectrometry. The results showed that the porous structure of biochar absorbed seven saturated hydrocarbons, thereby reducing the emission of asphalt VOCs. The third is flame-retardant action, which blocks the relationship between the asphalt binder and heat. Flame retardants absorb heat when heated, resulting in a decrease in the surface temperature of the asphalt binder, and then burn without heat exchange, thereby reducing the emissions of asphalt VOCs [23,24,25]. Li et al. [26] selected magnesium hydroxide, zinc borate, and ammonium polyphosphate as flame retardants, and used a titanate coupling agent to modify the surface functional groups of the flame-retardant raw materials. However, most flame retardants have the characteristics of low flame-retardant efficiency and large demand for addition, which seriously deteriorate the mechanical properties of polymer materials. The dosage of magnesium hydroxide powder needs to reach more than 60% to achieve the required flame retardancy, and a high dosage may have a great adverse effect on the physical properties of the asphalt binder.

After adding different emission modifiers to asphalt, the change in its performance has also attracted the attention of various researchers. Xiao et al. [27] incorporated activated carbon into asphalt binder as a VOCs inhibitor, and the results showed that activated carbon would reduce its permeability and ductility, but would increase its softening point, and the temperature stability at high temperature would be enhanced. Cui et al. [23] explored the possibility of adding styrene butadiene styrene (SBS) and activated carbon fillers to asphalt materials to develop environmentally friendly asphalt, and the results showed that the combined introduction of 4% styrene butadiene styrene (SBS) and 4% activated carbon can not only significantly reduce the speed and quantity of VOCs emission but also improve the high-temperature deformation resistance of asphalt. Li et al. [28] pointed out that the flame-retardant powder will adsorb the oily components of the asphalt, the penetration of the asphalt decreases monotonically with the increase of the flame retardant concentration, and the softening point of the modified asphalt tends to be stable, the high-temperature performance is improved, and the low-temperature performance decreases with the addition of flame retardants. Compared with other flame retardants, aluminum hydroxide powder has the greatest effect on asphalt ductility [29].

In summary, the means of reducing VOCs emissions in existing research is to add smoke suppressants (flame retardants, adsorbents, polymers and some new materials, etc.), and fix small molecules in asphalt smoke by physical or chemical methods, thereby reducing emissions, but the use of a certain additive alone will lead to a decline in some road performance of asphalt binder. Therefore, the method to effectively improve the effect of VOCs emission reduction and also ensure road performance is valuable to be investigated.

In this paper, a self-designed asphalt heating emission collection and detection device was proposed for an accurate evaluation, and the VOCs emission reduction performance of environmentally friendly modified asphalt after different types of modifiers was studied. The basic performance of modified asphalt was tested, and its VOCs inhibition effect on 70# virgin asphalt and its influence on basic performance was analyzed, and the optimal dosage was determined to provide a theoretical basis for the development of environmentally friendly modified asphalt with an applicable pavement performance.

## 2. Materials and Methods

### 2.1. Raw Materials

**Figure 1:**
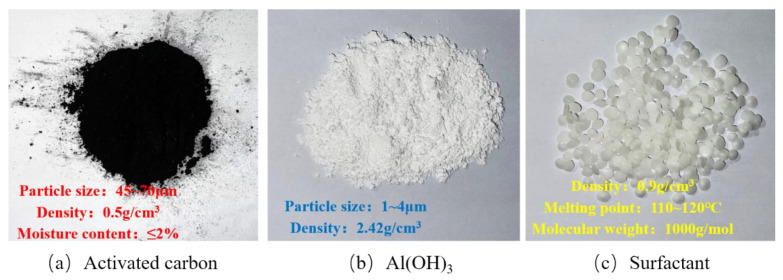
The schematics and properties of additives added for emission reduction; (**a**) Activated carbon, (**b**) Al(OH)_3_, (**c**) Surfactant.

In this investigation, 70# virgin asphalt was selected as the base asphalt. In order to investigate the modification effect of different additives on the virgin asphalt, three different additives are selected: activated carbon powder, Al(OH)_3_ powder, and surfactant. The main technical properties of base asphalt are shown in Table 1, and the specific parameters of different additives are shown in Figure 1.

**Figure 2:**
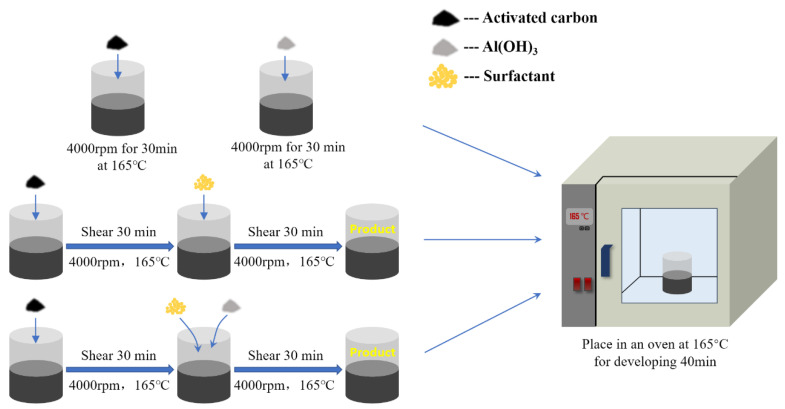
Preparation procedures of environmentally friendly modified asphalt.

The specific preparation process is shown in Figure 2. After the whole preparation process is completed, the prepared environmentally friendly modified asphalt is placed in an oven at 165 °C for 40 min, so that a large number of air bubbles generated due to the presence of air in the additive during the preparation of environmentally friendly modified asphalt are overflowed, which can also make the additive swells sufficiently with the bitumen.

### 2.2. Asphalt Heating Emission Evaluation Method

**Figure 3:**
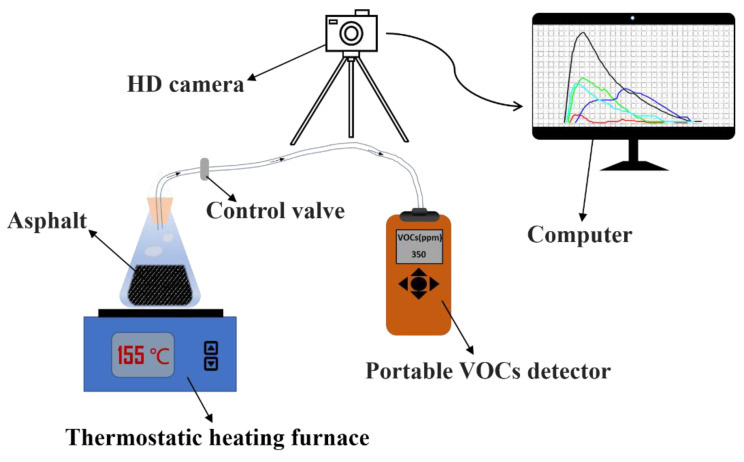
Asphalt heating-emission collection and detection device.

In this paper, a self-designed asphalt heating emission collection and detection device is used to detect the VOCs emission during the heating period. The collection and detection device consists of a constant temperature heating electric furnace, a conical flask, a portable VOCs detector, a control valve that controls the time when the heating emission enters the detector, a soft hose, a high-definition camera, and an electronic computer. The illustration of the device is shown in Figure 3.

Especially, the portable VOCs detector was customized from Aikesi Electronic Technology Co., Ltd., which has a detection range of 0~1000 parts per million (ppm) and a resolution of 1 ppm. Thus, the effective concentration of VOCs in this study was in the range of 0 to 1000 ppm.

Testing procedures are demonstrated as below: (1) put the conical flask containing the environmentally friendly modified asphalt into a 165 °C oven for half an hour; (2) take it out and place it on a constant temperature heating electric furnace and open the control valve; (3) after heating for 5 min, the detector starts to measure the VOCs concentration in the heating emission; and (4) the camera records the real-time change of VOCs concentration on the detector, which is convenient for subsequent data processing and analysis.

**Figure 4:**
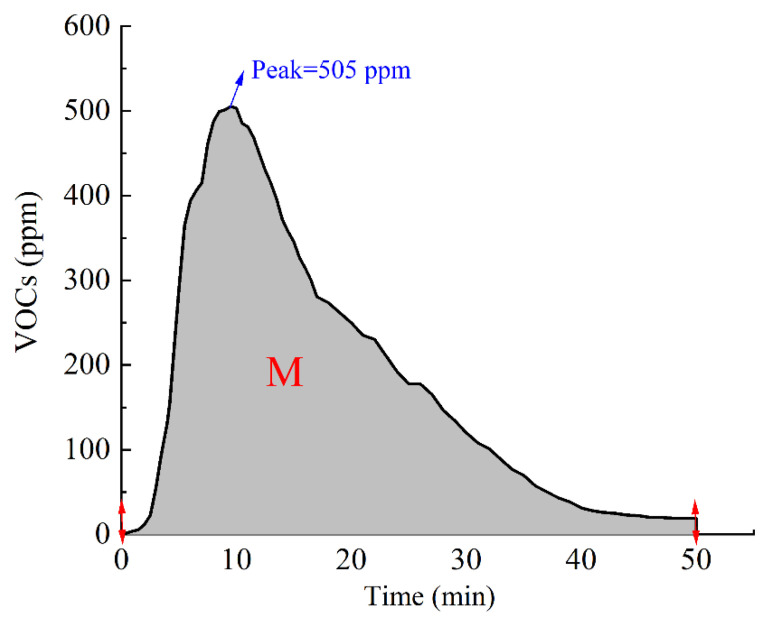
Illustration of VOCs emission M and peak value P.

To quantitatively characterize the effect of environment-friendly modified asphalt modified with different additives in inhibiting VOCs emission, as shown in Figure 4, the highest value of the recorded curve, the peak value P, and the area enclosed by the curve of VOCs concentration during the heating time is defined as M in Equation (1), which is the total emission of VOCs.

(1) $$M=\int_{t_{0}}^{t_{1}}V\left(x\right)dx\;\;\left(\min\cdot \mathrm{ppm}\right)$$

where *V*(*x*) is the curve of VOCs concentration changing with time, *t*_0_ is the time when the VOCs concentration is detected, and *t*_1_ is the time corresponding to the right endpoint of the VOCs concentration curve.

### 2.3. Test Plan

When heated at a constant temperature of 155 °C, the variation characteristics of VOCs concentration in heating emission of base asphalt and environment-friendly modified asphalt with different combinations of additives were detected and explored, as shown in Table 2. Among them, “C”, “S”, and “A” represent activated carbon, surfactant, and Al(OH)_3_, respectively, and the subscript numbers represent the specific dosage, such as “C_3_+S_3_”, which means 3% activated carbon and 3% surfactant.

Based on relevant research [18,26], the dosage of activated carbon was selected as 3%, 5%, and 7%. The dosage of surfactant was selected as 2.5%, 3%, and 3.5%. The content of Al(OH)_3_ was selected as 10%. The different combinations were designed to investigate the inhibitory effect of various dosage combinations on VOCs in heating emission at different temperatures, as shown in Table 2.

Considering the temperature of asphalt pavement construction in the field, three test temperatures were selected: 135 °C, 155 °C, and 175 °C.

The environment-friendly modified asphalt segregation test scheme is carried out in accordance with Chinese standard JTGE20-2011 [30]. The experiments in this paper are as follows: The prepared environment-friendly modified asphalt sample is put into an aluminum tube. Put the test sample at 163 °C for 48 h, then put the test sample into a cold environment to cool for 4 h, take two samples at the top and bottom of the aluminum tube for softening point test, and compare the softening point difference between the upper and lower parts to evaluate the segregation behavior.

## 3. Results and Discussion

### 3.1. Effects of Modifiers on VOCs Emission

**Figure 5:**
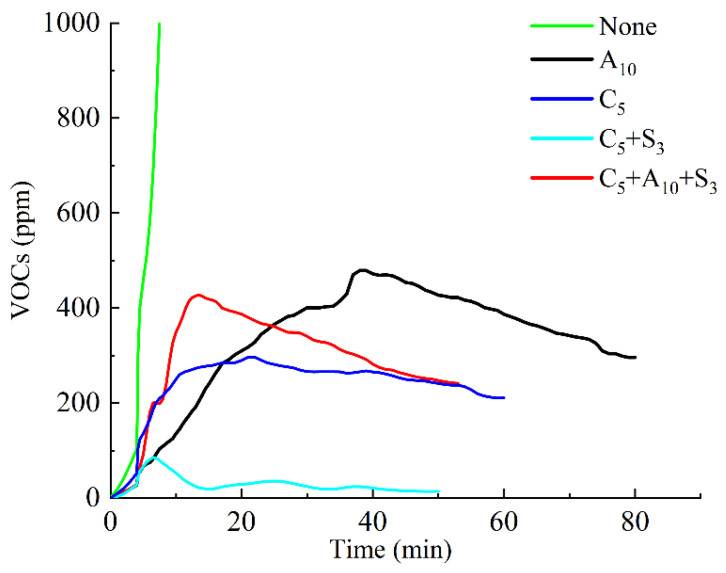
Asphalt VOCs emission curves under different modifier combinations.

The VOCs emission curve in the heating emission of environmentally friendly modified asphalt under different modifier combinations is shown in Figure 5, and the VOCs emission M and peak value P are shown in Table 3.

It can be seen that the VOCs concentration of the base asphalt quickly exceeded the 1000 ppm range within 10 min. With the increase of time, the VOCs concentration of the other four modified asphalts showed a trend of first increasing and then decreasing. After 50 min, the decreasing rate of the VOCs concentration tended to be stable.

Compared with the base asphalt, in the single-mixing scheme, the modified asphalt with A_10_ added reached a peak value of 489 ppm at the 39th min, and the VOCs emission was 26,276 min·ppm, while the VOCs concentration of the modified asphalt added with C_5_ increased steadily and reached a peak value of 298 ppm at the 21st min, and the VOCs emission decreased to 14,156 min·ppm. Obviously, the addition of C_5_ had the best inhibitory effect on the VOCs emission in the asphalt heating emission when it was added alone, and its VOCs concentration can reach the peak faster and the peak value was much smaller than that of the modified asphalt added with A_10_, and the VOCs emission was only 54% of the latter. This is because activated carbon is fine granular and has a porous structure. The specific surface area is generally as high as 500~700 m^2^/g, and it has a strong adsorption capacity for VOCs [18].

Compared with single blending, the modified asphalt added with C_5_+S_3_ had a more significant inhibitory effect on the emission of VOCs in heating emission. The peak value (86 ppm) appeared at 6.5 min and then decreased rapidly, and the VOCs concentration dropped to 29 ppm within 12.5 min, and then decreased. The VOCs concentration hovered around 23 ppm and eventually stabilized at 15 ppm. The VOCs emission was 1385 min·ppm, which is only one tenth of the modified asphalt with C_5_ added, and the effect was much better than other modified additive combinations.

For further consideration, the three-mixing of the additives showed that the addition of C_5_+A_10_+S_3_ modified asphalt did not have a better inhibitory effect on the emission of VOCs in the heating emission. Compared with the modified asphalt added with C_5_+S_3_, the peak and VOCs emissions were on the contrary. It increased several times, reaching 429 ppm and 15,297 min·ppm, respectively, even higher than the modified asphalt mixed with C_5_ alone. This may be because more than 90% of the surface area of activated carbon is concentrated in micropores, and it is the micropores that play an important role in the adsorption of activated carbon materials. Al(OH)_3_ particles are filled into the pores of activated carbon, which weakens the ability to adsorb VOCs.

### 3.2. The Effect of Activated Carbon Content

**Figure 6:**
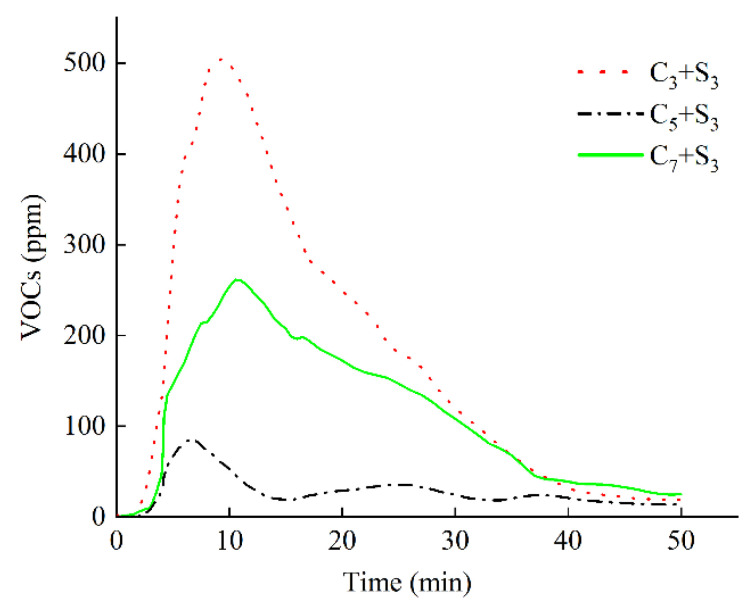
VOCs in heating emission of modified asphalt with different activated carbon content.

**Figure 7:**
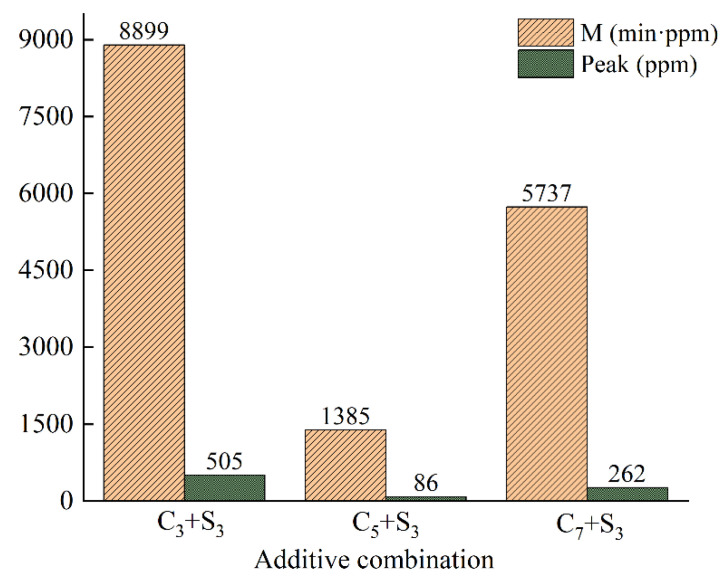
VOCs emission M and peak value P of environment-friendly modified asphalt under different activated carbon content.

The variation curve of VOCs concentration with time when the activated carbon content is changed is shown in Figure 6, and the VOCs emission M and peak value P are shown in Figure 7.

The environment-friendly modified asphalts with the addition of C_3_+S_3_, C_5_+S_3_, and C_7_+S_3_ showed a similar trend. With the increase of time, the concentration of VOCs first increased, then decreased, and then tended to be stable.

When the activated carbon content was 3%, the VOCs concentration reached a peak value of 505 ppm at the 9.5th min, and the VOCs emission was as high as 8875 min·ppm. Compared with 5%, the total VOCs emission growth rate and the peak growth rate were 542.5% and 487.2%, respectively. The inhibitory effect on VOCs in heating emission was greatly reduced. When the activated carbon content was 7%, the VOCs concentration reached a peak value of 262 ppm at the 11th min, and the VOCs emission was 5581 min·ppm; the inhibition effect on VOCs emission was better than that when the content of activated carbon was 3%, but not better than that when the content of activated carbon was 5%. This may be because when the amount of activated carbon is small, it has a significant adsorption effect on VOCs, and when extra activated carbon is added, agglomeration will occur, resulting in a relatively reduced specific surface area and reduced adsorption capacity.

### 3.3. The Effect of Surfactant Dosage

**Figure 8:**
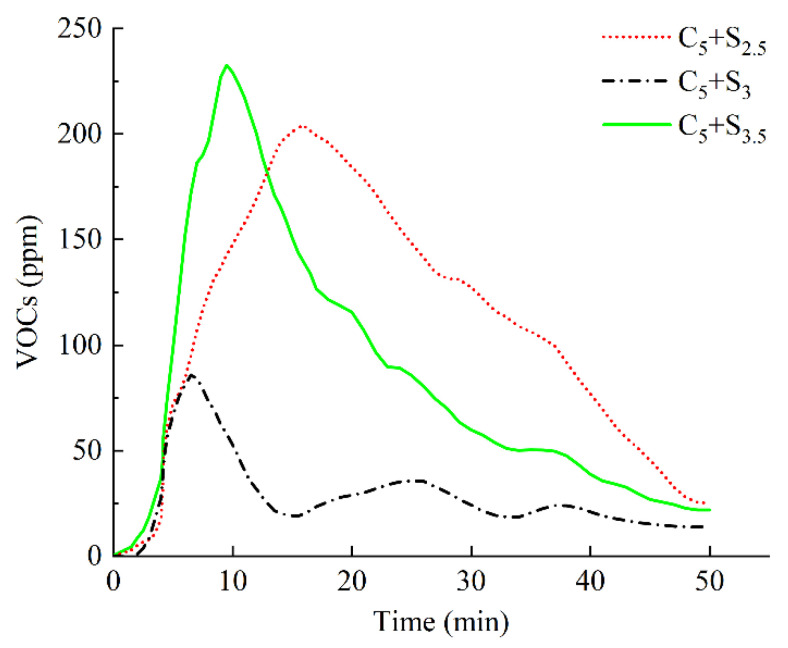
VOCs emission curve of environmentally friendly modified asphalt under different surfactant contents.

**Figure 9:**
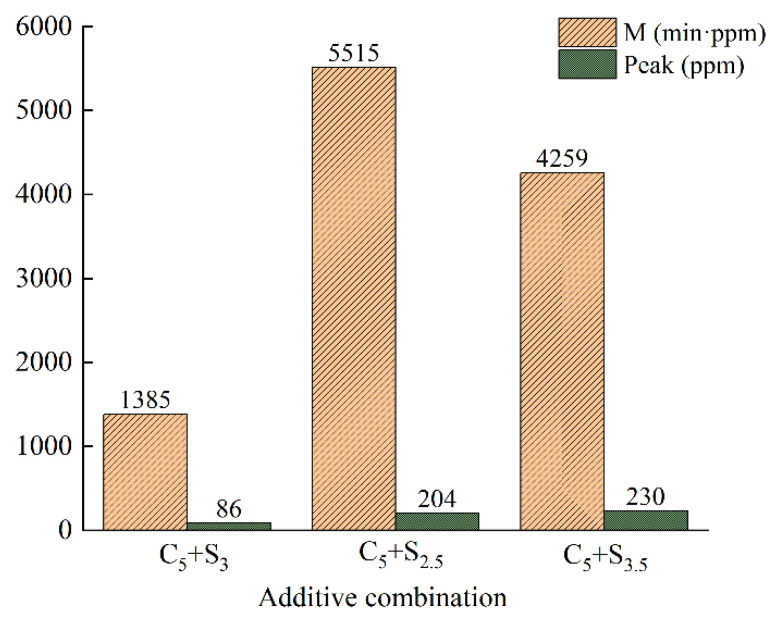
VOCs emission M and peak value P of environmentally friendly modified asphalt under different surfactant contents.

The variation curve of VOCs concentration with time when changing the surfactant dosage is shown in Figure 8, and the VOCs emission M and peak value P are shown in Figure 9.

The VOCs emission curve of modified asphalt with C_5_+S_3_ addition reached the peak (86 ppm) first, and the VOCs emission was only 1385 min·ppm.

When the surfactant dosage was 2.5%, the VOCs concentration reached a peak value of 204 ppm at the 16th min, and the VOCs emission was 5515 min·ppm. Compared with 3%, the total VOCs emission was about four times that of the modified asphalt with S_3_ added. When the surfactant dosage increased to 3.5%, the VOCs concentration reached a peak value of 230 ppm at the 10th min, and the VOCs emission was 4259 min·ppm. Compared with 3%, the total VOCs emission growth rate and the peak growth rate were 207.5% and 167.4%, respectively, and the inhibitory effect on VOCs in heating emission was greatly reduced.

### 3.4. The Effect of Temperature

**Figure 10:**
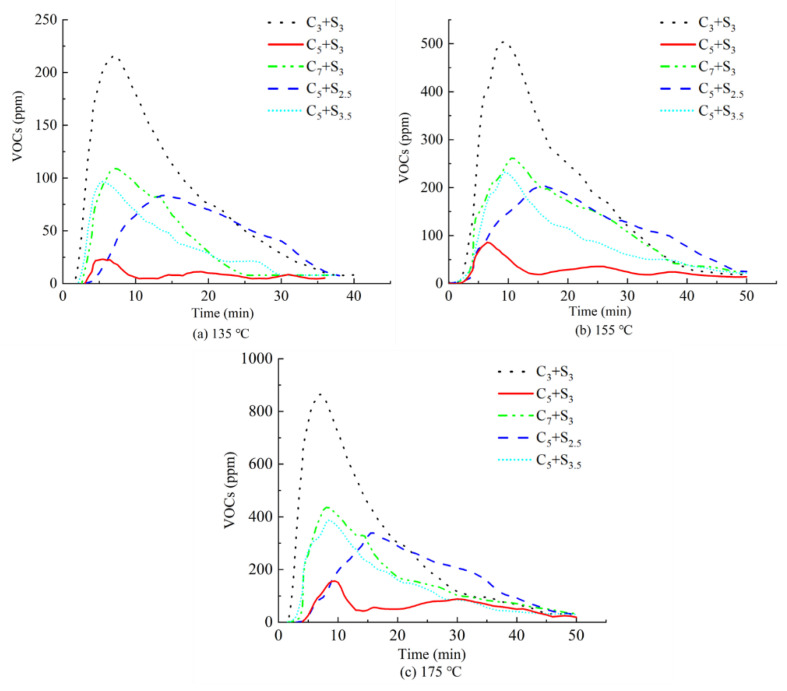
VOCs emission curve of environmentally friendly modified asphalt at different temperatures.

**Figure 11:**
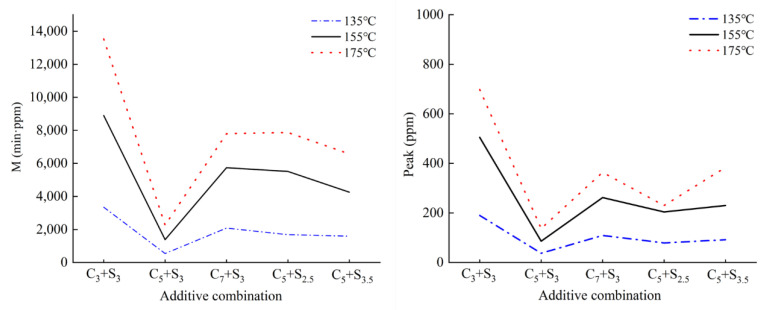
VOCs emission M and peak value P of environmentally friendly modified asphalt at different temperatures.

Figure 10 shows the VOCs emission curve in the asphalt heating emission when the environmentally friendly modified asphalt with different modifier combinations is heated at 135 °C, 155 °C, and 175 °C, and the VOCs emission M and peak value P are shown in Figure 11. The main factor affecting the release of asphalt heating emissions during construction is temperature. Studies have shown that [31] in the temperature range of 140 °C to 190 °C, the heating emission rate will increase by 2 times for every 12 °C increase in temperature. Figure 10 shows that the overall trend of the VOCs emission curves of the five environmentally friendly modified asphalts at different temperatures is consistent.

It can be seen from Figure 11 that the total VOCs emission M and the peak value of the environmentally friendly modified asphalt when heated at high temperature increased with the increase of the heating temperature, but the growth rate was different.

Taking the C_5_+S_3_ combination as an example, when the temperature increased from 135 °C to 155 °C, the growth rate of the total VOCs emissions and peak were 155.3% and 130.5%, respectively, while when the temperature increased from 155 °C to 175 °C, the total VOCs emissions and peak increased by are only 59.3% and 63.9%, which indicates that the increase of temperature is beneficial to improve the inhibitory effect of modifier on VOCs. Activated carbon purifies heating emissions mainly through physical adsorption. Its adsorption capacity is related to the surface area of the material, the size of the particles, the structure and distribution of pores, etc. The temperature has no obvious effect on the adsorption capacity of activated carbon. The surfactant selected in this paper is a white crystalline straight-chain aliphatic hydrocarbon mixture with a melting temperature between 110 °C and 120 °C. After melting, it can be completely dissolved in the asphalt as a saturated component. The higher the temperature, the faster and more complete the dissolution of the surfactant, which greatly improves the degree of its compatibility with the asphalt, making the inhibition effect on VOCs emissions more obvious [32].

### 3.5. Technique Properties of Environmentally Friendly Modified Asphalt

In order to evaluate the effect of different additive combinations on the basic properties of base asphalt, the penetration, softening point, and ductility were used to evaluate the conventional performance of base asphalt and environment-friendly modified asphalt.

**Figure 12:**
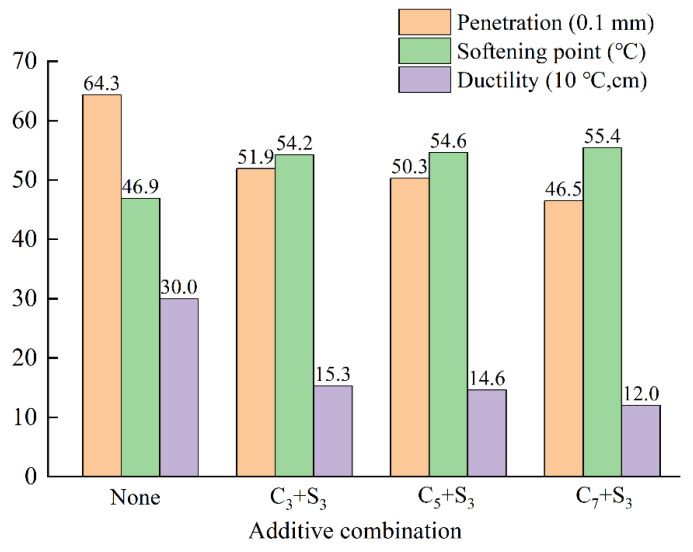
The changing law of the three major indexes of asphalt with the content of activated carbon.

From the data in Figure 12, it can be seen that when the content of activated carbon increased from 3% to 5%, the ductility decreased by 4.60%; when the content of activated carbon increased from 5% to 7%, the ductility decreased by 17.8%. The penetration and ductility of these three environmentally friendly modified asphalts decreased with the increase of activated carbon content, while the softening point increased with the increase of activated carbon content, which improved the high temperature stability of the asphalt. This is because the addition of hard materials restricts the movement of asphalt molecules to a certain extent, resulting in an increase in the consistency of asphalt and making it hardened and embrittled at low temperatures [33].

**Figure 13:**
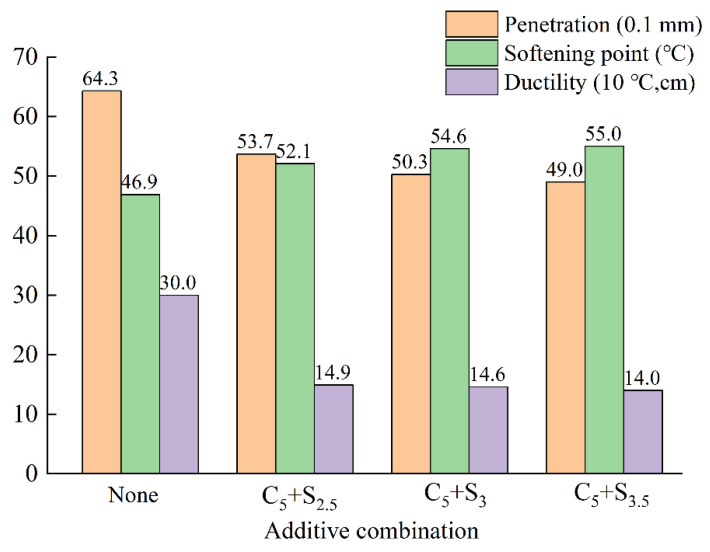
The changing law of the three indexes of asphalt with the different amount of surfactant.

Figure 13 shows that with the increase of the surfactant content, the penetration and ductility of the three environmentally friendly modified asphalts decreased; the softening point increased with the increase of the surfactant content, and the high-temperature stability also followed improved. This is due to the fact that since the melting point of the surfactant is 110~120 °C, the thermal degradation occurs at a temperature between 350 °C and 520 °C, and it has higher thermal stability than pure asphalt binder; at temperatures above 116 °C, it is completely miscible with asphalt binders, thereby improving the high-temperature stability of environmentally friendly modified asphalt. Surfactants will form lattice structures in asphalt at temperatures below their melting points. The formation of lattice structures prevents the movement of molecules in the modified binder, and this leads to a decrease in the ductility. When the content of surfactant increased from 2.5% to 3%, the softening point increased by 4.80%; when the content of surfactant increased from 3% to 3.5%, the softening point increased by 0.73%, the effect of improving the high-temperature stability of the environmentally friendly modified asphalt was not obvious, and it had an impact on the ductility of the asphalt. At the same time, considering the economic benefits, it is recommended that the dosage of surfactant is 3%.

### 3.6. Environmentally Friendly Modified Asphalt Segregation Test

It can be seen from the data in Table 4 that after the segregation test, the softening point of the lower part of the environmentally friendly modified asphalt was slightly lower than that of the upper part, but the difference was less than 2.5 °C. The phenomenon that the upper softening point was slightly larger may be because the relative density of the modifier is smaller than that of the base asphalt, and the internal molecular movement speed is accelerated after heating, resulting in the phenomenon of floating aggregation.

## 4. Conclusions

(1) A simple and self-designed asphalt heating emission collection and detection device is used to detect the concentration of VOCs in the heating emission of environmentally friendly modified asphalt with different additives. The combination of activated carbon and surfactant has the best inhibitory effect on the emission of VOCs in heating emissions.
(2) The overall trend of VOCs emission curves of environmentally friendly modified asphalt at different temperatures is consistent. The increase in temperature is beneficial to improving the inhibitory effect of surfactant on VOCs but has no obvious effect on the adsorption capacity of activated carbon.
(3) Compared with 70# base asphalt, the penetration of the environment-friendly modified asphalt under the combination of 5% activated carbon + 3% surfactant decreased by 1.4 mm, and the softening point increased by 7.7 °C, indicating that the high-temperature stability of the asphalt was improved and the resistance to deformation was enhanced. However, the ductility of the asphalt dropped to about 15 cm, and the addition of the modifier had a certain negative impact on the low temperature performance of the asphalt.
(4) Considering the inhibition effect on VOCs emission and the basic performance of environmentally friendly modified asphalt, it is suggested that the best additive combination is 5% activated carbon + 3% surfactant.

## Figures and Tables

**Table 1:** Technique properties of 70# virgin asphalt.

Technical Indexes	Results	Standard
Penetration, 0.1 mm	68	T0604-2011
Softening point, °C	46.5	T0606-2011
Ductility (10 °C), cm	25	T0605-2011
Dynamic viscosity (60 °C), Pa·s	198	T0620-2000
After TFOT		
Residual penetration ration (25 °C), %	68.2	T0604-2011
Mass change, %	0.04	T0609-1993
Residual ductility (10 °C), cm	7	T0605-2011

**Table 2:** Different additive combinations.

Additive Combination	Test Temperature (°C)
None	155
C_5_	155
A_10_	155
C_3_+S_3_	135 155 175
C_5_+S_3_	
C_7_+S_3_	
C_5_+S_2.5_	
C_5_+S_3.5_	
C_5_+A_10_+S_3_	155

**Table 3:** Asphalt VOCs emissions M and peak P under different modifier combinations.

Different Dosage Combinations	None	A_10_	C_5_	C_5_+S_3_	C_5_+A_10_+S_3_
M (min·ppm)	∞	26,276	14,156	1385	15,297
Peak (ppm)	≥1000	489	298	86	429

**Table 4:** Segregation test.

Sample	Softening Point (°C)		
	Lower Part	Upper Part	Difference
C_3_+S_3_	53.7	55.8	2.1
C_5_+S_3_	53.9	56.1	2.2
C_7_+S_3_	52.8	55.2	2.4
C_5_+S_2.5_	51.7	53.9	2.2
C_5_+S_3.5_	54.1	56.4	2.3

## Data Availability

All data in this paper can be obtained by contacting the corresponding author.
